# Enhancing Stability of High-Concentration β-Tricalcium Phosphate Suspension for Biomedical Application

**DOI:** 10.3390/ma16010228

**Published:** 2022-12-27

**Authors:** Kai-Wen Chuang, Yi-Chen Liu, Ramachandran Balaji, Yu-Chieh Chiu, Jiashing Yu, Ying-Chih Liao

**Affiliations:** Department of Chemical Engineering, National Taiwan University, Taipei 10617, Taiwan

**Keywords:** β-Tricalcium phosphate (β-TCP), aqueous suspension, polyacrylic acid, cellulose nanocrystals, ball milling, bone cement

## Abstract

We propose a novel process to efficiently prepare highly dispersed and stable Tricalcium Phosphate (β-TCP) suspensions. TCP is coupled with a polymer to enhance its brittleness to be used as an artificial hard tissue. A high solid fraction of β-TCP is mixed with the polymer in order to improve the mechanical strength of the prepared material. The high solid fractions led to fast particle aggregation due to Van der Waals forces, and sediments appeared quickly in the suspension. As a result, we used a dispersant, dispex AA4040 (A40), to boost the surface potential and steric hindrance of particles to make a stable suspension. However, the particle size of β-TCP is too large to form a suspension, as the gravity effect is much more dominant than Brownian motion. Hence, β-TCP was subjected to wet ball milling to break the aggregated particles, and particle size was reduced to ~300 nm. Further, to decrease sedimentation velocity, cellulose nanocrystals (CNCs) are added as a thickening agent to increase the overall viscosity of suspension. Besides the viscosity enhancement, CNCs were also wrapped with A40 micelles and increase the stability of the suspension. These CNC/A40 micelles further facilitated stable suspension of β-TCP particles with an average hydration radius of 244.5 nm. Finally, β-TCP bone cement was formulated with the suspension, and the related cytotoxicity was estimated to demonstrate its applicability for hard tissue applications.

## 1. Introduction

Numerous damages are inflicted on ligaments and bones from sports activities and accidents. The ability to recover from this damage is limited, and one solution for it is integrating artificial hard tissue. Bone cement is widely used to fabricate artificial bones. In the past, most orthopedic operations used polymethyl methacrylate (PMMA) to fill the cracks in the bone tissue due to its excellent mechanical and chemical properties [1]. A variety of research has shown the applicability of PMMA to bone cement. For example, Wekwejt et al. demonstrate a novel method to improve the bioactivity of PMMA bone cement without significant deterioration in mechanical properties of the bone cement by the addition of several components (nanosilver, cellulose, magnesium, and so on) [2]. By adding hydroxyethyl methacrylate (HEMA) and β-TCP in PMMA, Gao et al. improved the osteoconduction and enhanced the stability of bone cement [3]. Mixing calcium phosphates (CaPs) with polymeric bone cement systems also improves the mechanical properties and biocompatibility [4,5,6,7]. However, the hardening process of PMMA generates a great quantity of heat, which is quite harmful to the surrounding cells [8]. Hence, some research has introduced self-hardening bone cement mainly composed of CaPs and various polymers to fabricate artificial bone cement, which has a low setting temperature of about 45 °C [9,10,11,12]. Ślósarczyk et al. combined HAp(hydroxyapatite)-chitosan granules and α-TCP to fabricate the bone cement and show that small granules would decrease the setting time of bone cement for shorter operation procedures [13]. β-TCP is one such CaP that has excellent biocompatibility and a higher solubility than hydroxyapatite [14,15,16], which makes β-TCP an excellent candidate for artificial hard tissue formulation. Fang et al. fabricated PMMA bone cement modified by β-TCP and show that the modification improves the cell adhesion and proliferation of the acrylic bone cement [17]. Gallinetti et al. also fabricate a novel biphasic self-setting cement by a combination of α-TCP and β-TCP [18]. Nilsson et al. used the high solubility property of α-TCP to successfully fabricate bone cement with a compressive strength of up to 34 MPa [19]. Furthermore, the substituted TCP is also commonly used to enhance the metabolization of the bone cement. Sandra et al. used Sr-substituted α-TCP to fabricate artificial bone cement that showed great setting time and compressive strength [20]. The dispersion stability of β-TCP suspension has been shown to influence the uniformity of bone cement systems. Furthermore, the size of β-TCP also affects the mechanical strength and setting time [21,22]. Until now, the development of a fine-sized, high solid content, well-dispersed β-TCP suspension remains challenging for bone cement fabrication.

To fabricate a stable β-TCP suspension for the artificial hard tissue, the suspension should be well-dispersed and non-cytotoxic and possess low sedimentation velocity with small particles. To suspend β-TCP particles in water, dispersants should be added to modify the surface of particles. The added dispersant modifies the interface between the heterogeneous materials to enhance electrostatic repulsive interactions and physical steric barriers, which improves the suspension stability. The structure of a dispersant usually consists of long carbon chains and hydrophilic groups, such as sodium stearate [23] and polyacrylic acid [24]. The hydrophilic groups act as an anchor part to adsorb on the surface of the particles and make the interface highly charged to repel the particles to each other by classical DLVO theory. The long carbon chains are the chain segments that form steric barriers to prevent sedimentation [25]. Due to the interactions of the electrostatic repulsion and the physical steric barriers between the particles, the colloids can be deflocculated and suspended. 

In this research, a new formulation for bio-compatible β-TCP particle suspension in water was developed. The original particle size of β-TCP was large, greater than 1 μm, so that the effect of gravity was more influential than effect of Brownian motion [26]. Hence, ball milling was then adopted to grind the β-TCP into nano-scale particles for better suspension stability. A dispersing agent was also used to enhance the surface potential of the particles for better suspension stability. Moreover, to further slow down the particle sedimentation velocity, cellulose nanocrystals (CNCs), a bio-compatible material, were added as a thickening agent to increase the viscosity of the suspension for bone cement. The prepared β-TCP bone cement was then used to fabricate test samples. The related cytotoxicity was performed to demonstrate the applicability of the β-TCP suspension for bone cement. 

## 2. Materials and Methods

### 2.1. Chemicals

β-Tricalcium phosphate (Ca_3_(PO_4_)_2_, ≥98%, Tuzla, Istanbul, Turkey) is purchased from SUNUM e-Store. Polyacrylic acid (Dispex^®^ AA 4040, 99%, Ludwigshafen, Germany) was purchased from BASF. Cellulose nanocrystals (CNCs, Montreal, Quebec, Canada) were purchased from CelluForce. Poly(ethylene glycol) (PEG, MW 200, 99%, St. Louis, Missouri, USA) was purchased from Sigma-Aldrich. Citric acid monohydrate (≥99.0%, St. Louis, Missouri, USA) was purchased from Sigma-Aldrich. Cetyltrimethylammonium bromide (CTAB, ≥98%, Ward Hill, Massachusetts, United States) was purchased from Alfa Aesar. Cell Counting Kit-8 (CCK-8) was purchased from Enzo Life Sciences, Inc (New York, USA). Calcein AM (Live dye) and Ethidium homodimer I (dead dye) were purchased from Thermo Fisher Scientific (Waltham, Massachusetts, USA). Sodium chloride (≥99%, Phillipsburg, New Jersey, USA) was purchased from J.T Baker. Potassium chloride (≥99%, St. Louis, Missouri, USA) was purchased from Sigma-Aldrich. Sodium phosphate dodecahydrate (≥97%, Branchburg, New Jersey, USA) was purchased from ACROS. Potassium phosphate (≥99%, Phillipsburg, New Jersey, USA) was purchased from J.T Baker. Phosphate buffer saline (PBS) was fabricated by adding 8 g of sodium chloride, 0.2 g of potassium chloride, 1.44 g of sodium phosphate dibasic, and 0.245 g of potassium phosphate monobasic to 800 mL of distilled water. The solution was adjusted to the desired pH of 7.4. Then, distilled water was added to the solution until the volume was 1 L.

### 2.2. Formulation of β-TCP Nano-Suspension 

**Figure 1:**
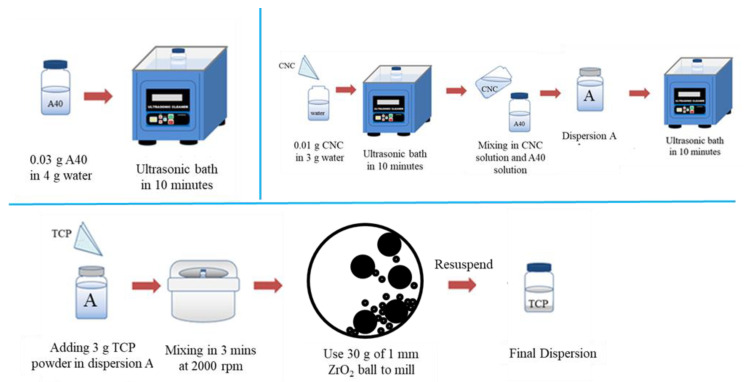
Schematic illustration of the formulation of dispersion.

A schematic diagram for the formulation of β-TCP nano-suspension is shown in Figure 1. First, 0.03 g of A40 was added to 4 g DI water and 0.01 g CNC in 3 g DI water. Both A40 and CNC solutions were ultrasonicated for 10 min and then mixed together, which formed dispersion A. Upon sonicating the dispersion A for 10 min, 3 g of β-TCP was added into dispersion A. Later, after the planetary centrifugation at 2000 rpm (3 min), the solution was subjected to ball milling with 30 g zirconium balls, whose parameters were 700 rpm, 5 min runtime, 5 min pause time, and 7 cycles. The solution was separated from zirconium balls by 140-mesh sieves, and we used a small amount of DI water (about 40–50 mL) to wash out all suspension adsorbed on zirconium balls. The precipitate was collected after centrifugation at 6000 rpm for 6 min, and its solid weight was calculated by weighing and drying. Assuming the A40 and CNC was all washed out during centrifugation, we repeated the fabrication steps of dispersion A to re-disperse the sedimentation, whose weight ratio was 10% β-TCP, 0.3% A40, and 0.1% CNC.

### 2.3. Formulation of β-TCP Bone Cement

The β-TCP sediment, which was collected by centrifugation as explained in Section 2.2, was dried and mixed with 10 wt % PEG and 20 wt % citric acid in DI water with a liquid-to-powder ratio of 0.7 mL/g. PEG was selected for its curability [27], and citric acid was chosen as the setting liquid for its non-toxicity [28]. After mixing, the cement was dried under an ambient environment for 30 min.

### 2.4. Cytotoxicity Test

The cytotoxicity of the material was determined using a CCK-8 assay. To obtain the extract solution, materials were cut into 8 mm by 8 mm by 3 mm cuboids and exposed to UV light for 1 h to ensure complete sterilization. Then, immersed cuboids of materials were placed into 1 mL fresh culture medium and incubated in a 37 °C incubator for 4, 24, and 48 h. The L929 cells were seeded at a density of 5 × 10^4^ cells per well with 1 mL extract solution in a 48-well plate and let stand in a 37 °C incubator for 4, 24, and 48 h. After 4, 24, and 48 h, the extract solution was replaced by fresh phosphate-buffered saline (PBS) containing CCK-8 reagent. Then, the absorbance of the cells’ media was detected at 450 nm. However, for the control group in this test, the L929 cells were cultured in fresh medium for 4, 24, 48 h.

Live/Dead assays were performed as cytotoxicity tests and cell viability qualitatively. To obtained the extract solution, materials were cut into 8 mm by 8 mm by 3 mm cuboids and exposed to UV light for 1 h to ensure complete sterilization. Then, cuboids of materials were immersed into 1 mL fresh culture medium and incubated in a 37 °C incubator for 48 h. The L929 cells were seeded at a density of 5 × 10^4^ cells per well with 1 mL extract solution in a 48-well plate and let stand in a 37 °C incubator for 48 h. After 48 h, Live/Dead was performed by washing the cells with PBS and staining the cells with Calcein AM (Live dye) and Ethidium homodimer I (Dead dye) diluted 1:500 in PBS for 10 min at 37 °C. Then, the cells on the well plate were observed with fluorescence microscopy directly. However, for the control group in this test, the L929 cells were cultured in fresh medium for 48 h.

### 2.5. Device and Characterization

Ultrasonication bath (DC150H, DELTA, New Taipei, Taiwan) and planetary centrifugal mixer (THINKY MIXER ARE-310, THINKING, Laguna Hills, California, USA) was used to make the solution well-mixed. Ball milling was performed using a PULVERISETTE 7 premium line (FRITSCH, Idar-Oberstein, Germany). The universal high-speed centrifuge was HERMLE N.Z326 (HERMLE, Gosheim, Germany). Scanning Electron Microscope (SEM; Nova NanoSEM 230, FEI, Hillsboro, Oregon, USA) was used to analyze the size and shape of β-TCP. X-ray Diffractometer (XRD; SmartLab SE, Rigaku, The Woodlands, Texas, USA) was used to confirm the powder composition and stability. A scan range from 10° to 60° was used with a scan rate of 30°/min. Size distribution and zeta-potential were estimated by Malvern Zetasizer Nano (Malvern, Malvern, UK). The size distribution and zeta-potential were measured 20 times per round. Viscosity was examined by Brookfield DV-III Ultra (Brookfield, Middleboro, Massachusetts, USA). The thermogravimetric analysis (TGA, SDT 650, TA Instrument, New Castle, Delaware, USA) of the TCP suspension is performed in nitrogen condition at a heating rate of 10 °C/min. 

## 3. Results and Discussion

### 3.1. Physical Characterizations of β-TCP

**Figure 2:**
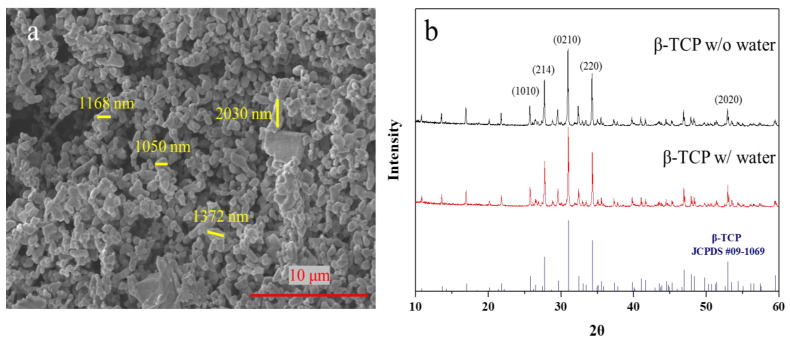
(**a**) X-ray diffraction analysis result of β-TCP powder (black for origin powder; red for soaked powder) and standard β-TCP peak. (**b**) Scanning electron microscope image of original β-TCP particles.

Tricalcium phosphate (TCP) is often used in fabricating the bone cement for medical use. Its structure is divided into α-type and β-type, and the TCP used in this study is β-type. Scanning electron microscopy (SEM) was first used to investigate and to understand the morphology of the β-TCP powder. As shown Figure 2a, the powder aggregation of β-TCP was quite serious. Most of the particles were above 1 μm, with some smaller particles of ~600 nm in diameter.

To confirm the crystalline structures of TCP, a X-ray diffractometer (XRD) was used. As shown in Figure 2b, the characteristic peaks of β-TCP are consistent with its lattice planes (Figure 2a) [29]. The crystal diffraction pattern confirms that the TCP powder is the β type. To ensure the quality of the TCP particles (α-TCP hydrolyzed in a few days [30]), the particles were stored in water for a couple days and the crystalline structures were checked again. The spectra of the received β-TCP in water show that β-TCP structures remained unchanged after β-TCP was immersed and stirred in water for three days. This result indicates that the particles were composed of high-purity β-TCP. 

### 3.2. Influences of Dispersant on Ink

To improve the suspension stability of the β-TCP, a dispersing agent is needed to modify the β-TCP surface. The common dispersant can be categorized as cationic or anionic. To understand the suitable polarity of the dispersants, two different agents were tested: anionic dispersant (A40) and cationic cetyltrimethylammonium bromide (CTAB). In Figure S1, it can be found that the sedimentation speed of β-TCP particles was lower in the suspension with A40. Therefore, A40 was chosen to disperse the agglomerated β-TCP nanoparticles to improve the suspension stability. A40 is an ammonium polyacrylate (NH_4_PAA) solution that is often used as a dispersant for colloidal suspension. It has low viscosity and is suitable for use in aqueous systems, and it has FDA certification. When NH_4_PAA dissolves in water, it dissociates to form negatively charged carboxylate anions (RCOO^−^) and ammonium (NH_4_^+^) according to formula (1), and the RCOO^−^ ions in water form RCOOH and OH^−^, making the solution weakly alkaline as shown in formula (2) [31]. A40 is physically adsorbed on the surface of TCP particles, and long carbon chains, COO^−^ ions, are connected to the surface of TCP, so that there are steric barriers on particles’ surface due to long carbon chains and large surface potential due to multiple COO^−^ ions. Hence, A40 would disperse the agglomerated β-TCP nanoparticles to improve the suspension of the ink.

RCOONH_4_ → RCOO^−^ + NH_4_^+^ (1)

RCOO^−^ + H_2_O → RCOOH + OH^−^ (2)

**Figure 3:**
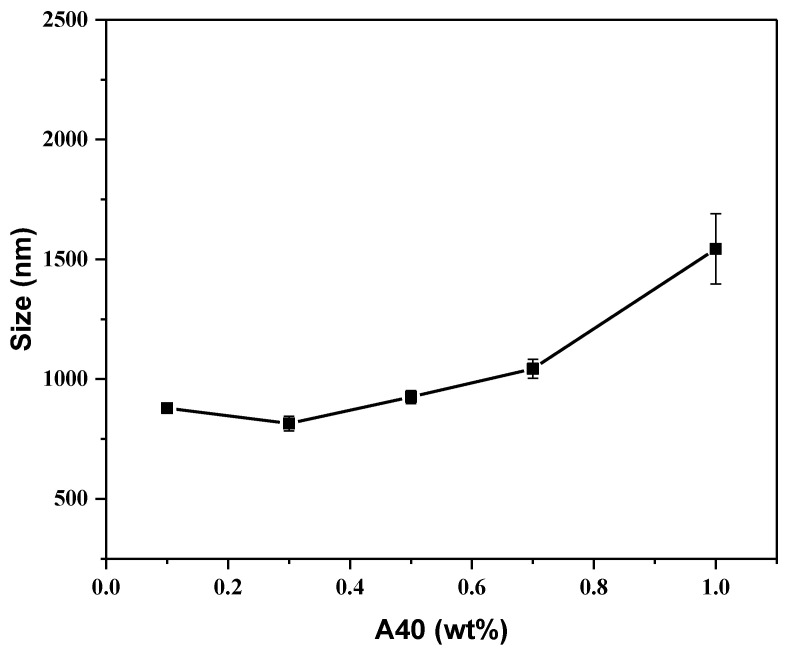
Size analysis for β-TCP suspension in different A40 concentrations.

From Figure S2, we observed the difference between β-TCP suspension with and without A40. Although the A40 efficiently suspended β-TCP particles, the particles were still easily suspended. Hence, we adjusted the optimal addition ratio of TCP powder and A40 dispersant to improve the suspension phenomenon. Through dynamic light scattering (DLS), the distribution of the dispersed particles could be observed. From Figure 3, it can be seen that the lowest dispersed particle size was 814.7 nm at 0.3 wt % A40, but the precipitation phenomenon was still intense such that it hardly formed a stable suspension. When A40 exceeded 0.3 wt %, β-TCP had excessive polymers on the surface, which were entangled with each other to cause flocculation and precipitate. It can be seen that the dispersed particle size rose rapidly. When A40 was less than 0.3 wt %, there was too little A40, so the A40 could not completely wrap the β-TCP particles, resulting in the incompletely effective surface modification of the β-TCP surface. The experimental results show that the addition of the dispersant has adverse effects. Too little dispersant (<0.3 wt %) is not enough to coat the β-TCP particles and provide sufficient repelling force, while too much dispersant (>0.3 wt %) makes the long chains of A40 entangled with each other, resulting in aggregation and further precipitation. Hence, 0.3 wt % A40 is chosen for the following steps.

### 3.3. Influence of Ball Milling β-TCP

**Figure 4:**
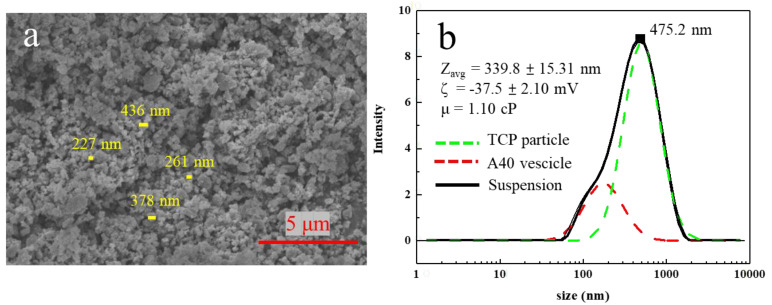
(**a**) SEM image of original β-TCP particles. (**b**) Size distribution for β-TCP suspension with 0.3 wt% A40 after ball milling and resuspension.

From the above experiments and SEM images, it was inferred that the original particle size of β-TCP was large (>1 μm), and the β-TCP particles could not be effectively dispersed and suspended even after a dispersant was added. Therefore, ball milling was adopted to break the β-TCP particles through the high shearing force and make the particle size smaller to facilitate the subsequent preparation of suspension ink. When using wet grinding, water would flow into the cracks appearing on the particles, which would act as a barrier. After the impact and extrusion of the grinding medium, the cracks gradually enlarge, and finally, the bulk particles are crushed. While the cracks of dry grinding are not filled with substances, and the cracks may be squeezed smaller during the ball milling process, making the ball milling effect worse. If no dispersant is added, the dispersed particles are aggregated into agglomerates under the action of the Van der Waals force, which reduces the impact of ball milling. With the addition of dispersant, the adsorbed A40 surface layer on the particles would repel each other due to the three-dimensional barrier and surface potential, which prevents particles from aggregating again during ball milling. At this time, the impact of the ball milling beads again grind the particles into smaller particles. The dispersant is re-coated, and then the ball-milling beads are re-milled, and so on. Finally, β-TCP particles that are much smaller than the original particles can be milled [32,33]. Hence, the dispersant and water are added before the ball milling process. Figure 4a shows the SEM image of β-TCP after ball milling. It can be found that the β-TCP particles after ball milling are much smaller than those before ball milling.

After ball milling, most of the β-TCP suspension will stick on the zirconium beads, so the β-TCP suspension on the zirconium beads was washed with a small amount of DI water and then sieved. The addition of DI water caused A40 to desorb from the β-TCP surface, resulting in the surface of β-TCP no longer having steric barriers and additional surface potential. Hence, particles aggregate and precipitate. Furthermore, because of the addition of DI water, the re-suspending method was used to suspend the ball-milled β-TCP particles. Using high-speed centrifugation, A40 could be removed from the surface of β-TCP. After separating the sediment and supernatant, we measured the weight and solid content of the sediment to calculate the required amount of water and A40 to re-suspend. For the final suspension of 10% β-TCP and 0.3% A40, it can be seen from Figure 4b that the average dispersed particle size of this suspension reached 339.8 nm, and the suspension could still be stably suspended after one day without obvious stratification. However, the main peak fell at 475.2 nm and the particle size distribution is not small enough and had two peaks, but this result proves that the simultaneous use of dispersant and ball milling could effectively reduce the dispersed particle size and stably suspend.

### 3.4. Influence of CNC towards β-TCP Suspensions

**Figure 5:**
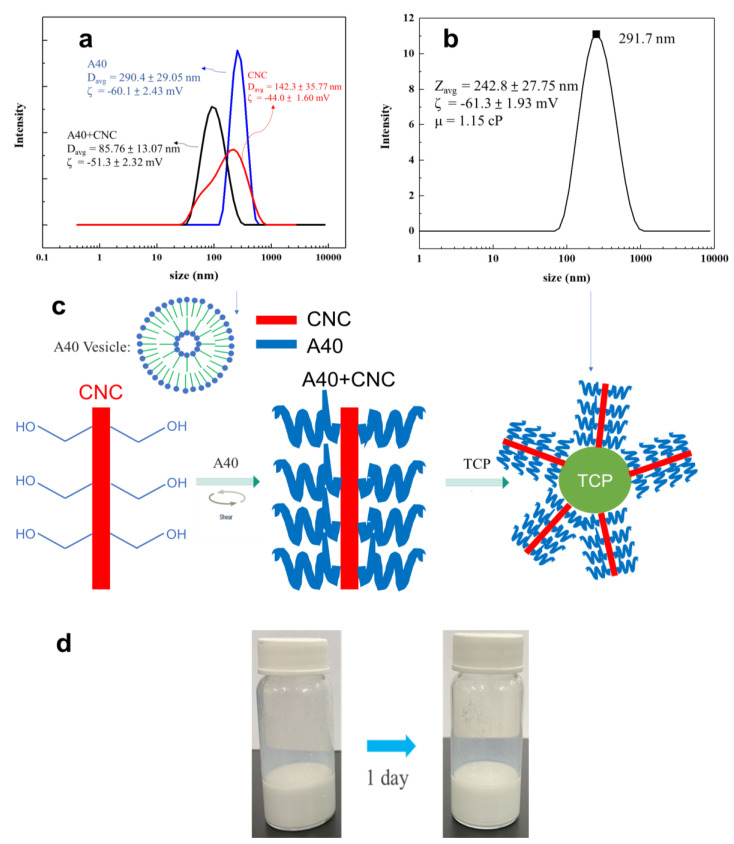
(**a**) Result of the size distribution of 0.3 wt % A40/ 0.1 wt % CNC in water. (**b**) Size distribution for β−TCP suspension with 0.3 wt % A40, 0.1 wt % CNC after ball milling and resuspension. (**c**) Schematic diagram of the interaction between CNC, A40, and TCP. (**d**) Image of β−TCP suspension after one day.

The viscosity is inversely proportional to the sedimentation velocity. Hence, the CNC was added to increase the viscosity to slow down the sedimentation velocity, resulting in better suspension stability. Cellulose nanocrystals were formed by hydrolyzing natural fibers. They have high biodegradability and can increase the viscosity of the liquid to increase the shear force between the liquid and the nanoparticles, making the nanoparticles more stably suspended in the liquid [34,35,36,37]. Later, we used 10% TCP, 0.3% A40, and 0.1% CNC to make TCP suspension. From Figure S3, it could be found that an effective and stable suspension of TCP aqueous solution could not be made without ball milling. Therefore, the ball milling and re-suspending process were repeated again and the CNC was also added for dispersal. It was observed that its dispersing effect was much better than that without ball milling. Even after one day, the upper and lower layers were not separated, as shown in Figure 5d. From Figure 5b, it was surprisingly observed that the average dispersed particle size reached 242.8 nm.

**Figure 6:**
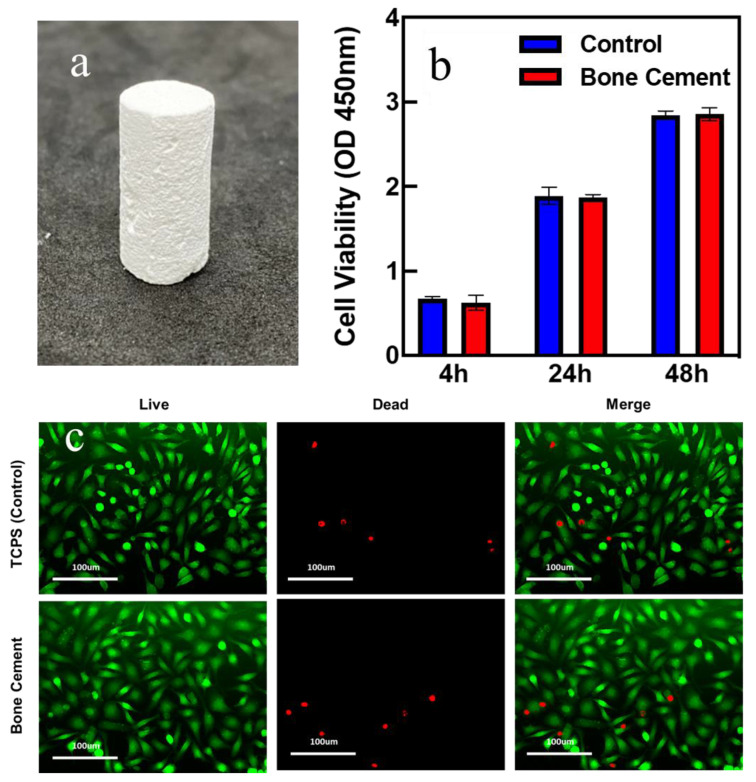
(**a**) Image of the hardened β-TCP bone cement. (**b**) The cell viability of the bone cement and control group. (**c**) Live(green)/dead(red) assay of the bone cement and control group.

Because the dramatic decrease in dispersed particles, the dissolution phenomenon of A40 and CNC was studied. Using DLS, it could be seen that the dispersed particle size of A40 in water was 290.4 nm, from which it could be determined that 0.3 wt % A40 formed vesicles when dissolved in water, making it less likely to adsorb on TCP particles. Besides the size distribution of dissolved A40, the vesicles can be observed from Figure 4b at approximate 200 nm. The average particle size of CNC in water was 142.3 nm and had a wide particle size distribution with multiple peaks. After we added A40, it became a single peak, and the average particle size was 85.76 nm, which represents a significant decrease. This indicates that, by adding A40, we could increase the steric barriers on the surface of CNC so that the aggregated CNC particles were separated and size was reduced. When the stick-shaped CNC is connected with A40, it should form a long stick connected with multiple A40 dispersants [38,39], as shown in Figure 5c. Finally, the multi-branched sticks were adsorbed to the surface of the TCP, forming stronger steric barriers, so as to more effectively disperse the TCP. In Figure 6, no peaks of A40 and CNC are seen at 90, which means that the multi-branched sticks combined with A40 and CNC were connected to TCP. Therefore, the surface of TCP has a strong stereoscopic barrier and surface potential. The absolute value of the zeta potential needs to be greater than 30 mV to be stable, and the zeta-potential of our suspension reached −61.3 mV, so it could be determined that this liquid is a very stable suspension in terms of surface potential.

To present the practical applicability of β−TCP ball-milled suspension, β−TCP bone cement was fabricated to present the applications of the β−TCP suspension. Figure 6a shows an example demonstrating the artificial bone cement product. Furthermore, the cytotoxicity was examined to prove that cement does not harm cell cultivation. From Figure 6b, it can be seen that the cell viability of bone cement increased as the time changed and was quite close to that of the control group. To perform the cytotoxicity tests qualitatively, the live/dead assay was also examined. From Figure 6c, after cultivation for 48 h, the majority of the cells on the bone cement were viable with only a few dead cells in the sample, indicating the bio-compatibility of the prepared bone cement. Furthermore, the number of viable cells in the bone cement sample was close to that on the control samples from the images. The above experiments indicated that the bone cement is harmless to cell growth.

## 4. Conclusions

In this research, a novel formulation was developed to fabricate β-TCP stable suspension to apply in bone cement fabrication. The TCP used in this research is stable in water without hydrolysis for 3 days and is a high-purity powder with an average size of ~1 μm used for examining the XRD pattern and SEM images. With the addition of A40 dispersant, steric barriers were formed between the TCP particles, and electrostatic repulsion on the surface of TCP enhanced the suspension stability. To further enhance the suspension stability, a wet ball-milling method was adopted to decrease the average particle size down to ~300 nm. CNC was selected to increase the viscosity and decrease the sedimentation speed. Furthermore, CNC could disperse the A40 vesicles, which made A40 unable to modify the TCP surface. After the addition of CNC/A40 and the ball-milling process, a suspension with a low average particle size of 242.8 nm and a high zeta-potential of −61.3 mV was successfully formulated. Furthermore, β-TCP bone cement was made to show the applicability of this formulation, and it is bio-compatible with cell cultivation. This formulation demonstrated here shows great improvement in the β-TCP suspension stability and is applicable for artificial hard tissue fabrication.

## Data Availability

Data are contained within the article.

## Supplementary Materials

The following supporting information can be downloaded at: https://www.mdpi.com/article/10.3390/ma16010228/s1, Figure S1: Sedimentation of β-TCP suspension with different dispersants. Figure S2: Comparison of β-TCP suspension with and without A40. Figure S3: Image of β-TCP suspension with A40 and CNC but without ball milling. Figure S4: TGA analysis of β-TCP suspension after resuspended [40].
