# Supplementary material for: Enhancing Stability of High-Concentration β-Tricalcium Phosphate Suspension for Biomedical Application
Source: Materials (Basel). 2022 Dec 27;16(1):228. doi: 10.3390/ma16010228 (PMC9822431; doi:10.3390/ma16010228)
Supplement: Supplementary file 1 [file materials-16-00228-s001.zip › materials-2012497-supplementary.pdf]

materials

Supporting Information for:

Enhancing Stability of High-Concentration β-Tricalcium Phosphate Suspension for Biomedical Application

Kai-Wen Chuang, Yi-Chen Liu, Ramachandran Balaji, Yu-Chieh Chiu, Jiashing Yu and Ying-Chih Liao *

Department of Chemical Engineering, National Taiwan University, Taipei 10617, Taiwan

* Correspondence: liaoy@ntu.edu.tw

Contents:

I. Sedimentation of β-TCP suspension with different dispersants

II. Comparison of β-TCP suspension with and without A40

III. Image of β-TCP suspension with A40 and CNC but without ball milling

IV. TGA analysis of β-TCP suspension after resuspended

I. Sedimentation of β-TCP suspension with different dispersants

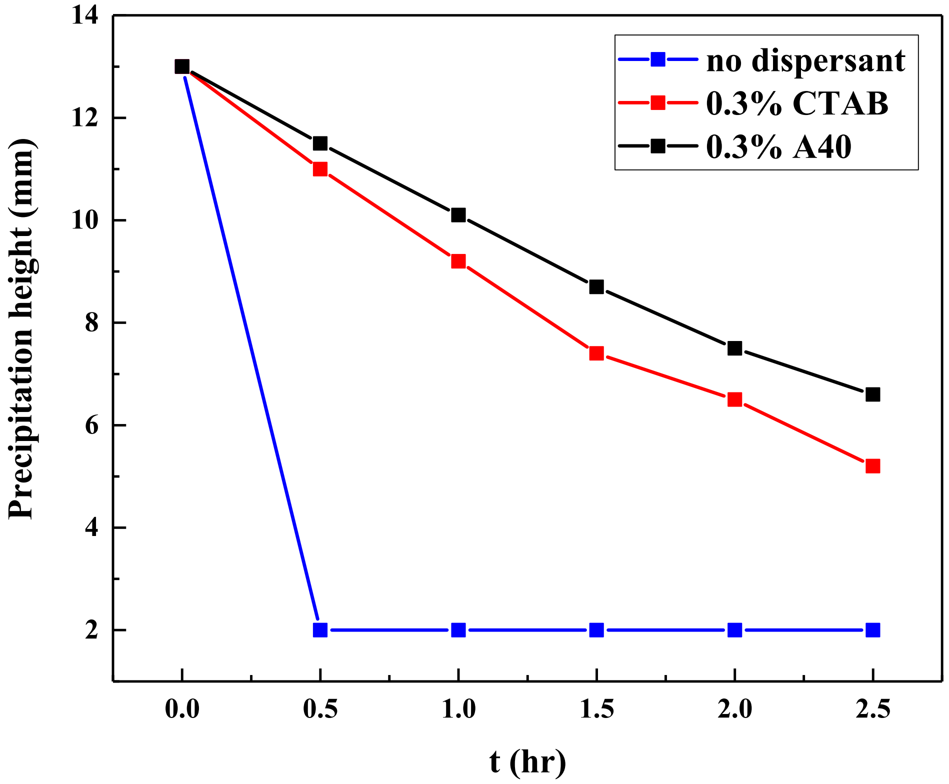

Figure S1. Sedimentation speed of β-TCP suspension with dispersants of different electronic property.

II. Comparison of β-TCP suspension with and without A40

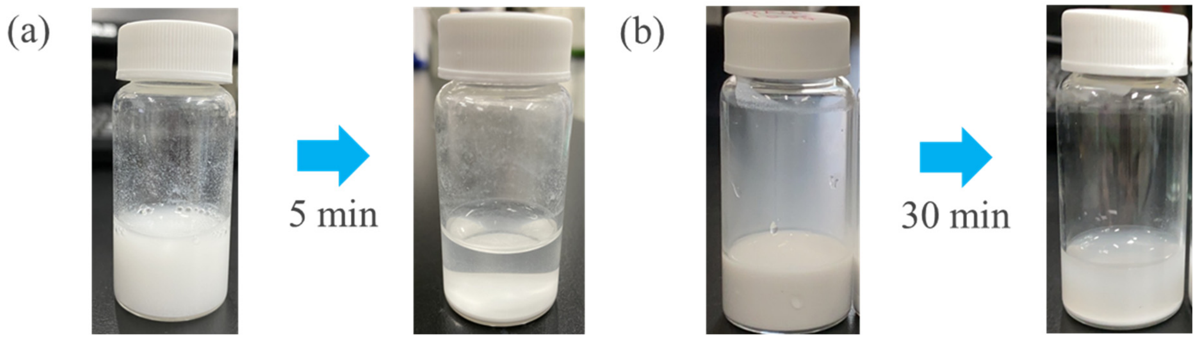

Figure S2. Comparison of suspension before and after adding dispersant (a) no dispersant (b) 0.3 wt% A40.

III. Image of β-TCP suspension with A40 and CNC but without ball milling

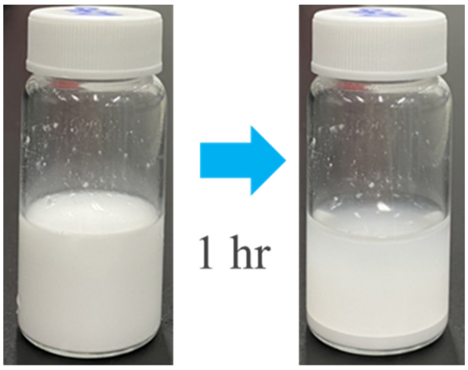

Figure S3. β-TCP suspension with 0.3 wt% A40 and 0.1% CNC without ball milling.

IV. TGA analysis of β-TCP suspension after resuspended

To understand the degradation of the TCP suspension, the TGA analysis of β-TCP is investigated. In **Figure S4(A)**, most of the water in the suspension evaporates at 70 °C and the residual weight remains almost consistent from 100 to 700 °C. However, when the Figure S4(A) is magnified to Figure S4(B), it shows that the residual weight decreases slightly from 100 to 500 °C. Because the TCP powder would not degrade at that temperature[40], it can be concluded that the decrease of the residual weight is due to the degradation of the dispersant (A40 and CNC).

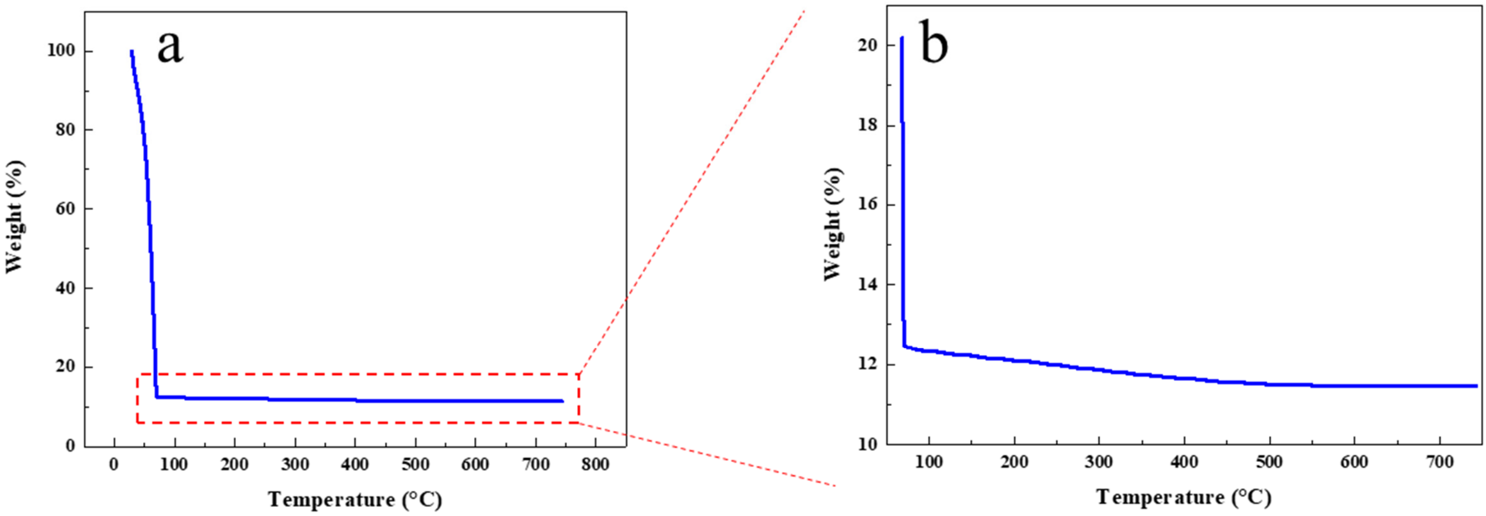

Figure S4. (A) TGA analysis of β-TCP suspension (B) magnified chart from TGA analysis.

Reference

40. Kumar, B. S.; Hemalatha, T.; Deepachitra, R.; Raghavan, R. N.; Prabu, P.; Sastry, T., Biphasic calcium phosphate–casein bone graft fortified with Cassia occidentalis for bone tissue engineering and regeneration. *Bulletin of Materials Science* **2015,** 38, (1), 259-266.
